# Vector Competence of American Mosquitoes for Three Strains of Zika Virus

**DOI:** 10.1371/journal.pntd.0005101

**Published:** 2016-10-26

**Authors:** James Weger-Lucarelli, Claudia Rückert, Nunya Chotiwan, Chilinh Nguyen, Selene M. Garcia Luna, Joseph R. Fauver, Brian D. Foy, Rushika Perera, William C. Black, Rebekah C. Kading, Gregory D. Ebel

**Affiliations:** Department of Microbiology, Immunology and Pathology, Colorado State University, Fort Collins, Colorado; INDEPENDENT RESEARCHER, UNITED STATES

## Abstract

In 2015, Zika virus (ZIKV; *Flaviviridae; Flavivirus*) emerged in the Americas, causing millions of infections in dozens of countries. The rapid spread of the virus and the association with disease outcomes such as Guillain-Barré syndrome and microcephaly make understanding transmission dynamics essential. Currently, there are no reports of vector competence (VC) of American mosquitoes for ZIKV isolates from the Americas. Further, it is not clear whether ZIKV strains from other genetic lineages can be transmitted by American *Aedes aegypti* populations, and whether the scope of the current epidemic is in part facilitated by viral factors such as enhanced replicative fitness or increased vector competence. Therefore, we characterized replication of three ZIKV strains, one from each of the three phylogenetic clades in several cell lines and assessed their abilities to be transmitted by *Ae*. *aegypti* mosquitoes. Additionally, laboratory colonies of different *Culex* spp. were infected with an American outbreak strain of ZIKV to assess VC. Replication rates were variable and depended on virus strain, cell line and MOI. African strains used in this study outcompeted the American strain *in vitro* in both mammalian and mosquito cell culture. West and East African strains of ZIKV tested here were more efficiently transmitted by *Ae*. *aegypti* from Mexico than was the currently circulating American strain of the Asian lineage. Long-established laboratory colonies of *Culex* mosquitoes were not efficient ZIKV vectors. These data demonstrate the capacity for additional ZIKV strains to infect and replicate in American *Aedes* mosquitoes and suggest that neither enhanced virus replicative fitness nor virus adaptation to local vector mosquitoes seems likely to explain the extent and intensity of ZIKV transmission in the Americas.

## Author Summary

The mechanisms contributing to the explosive nature of the current ZIKV outbreak in the Americas are poorly understood. Therefore, we characterized the replication of three strains, one from each phylogenetic clade of ZIKV and evaluated virus strain differences in transmission efficiency by American mosquitoes. Our results suggest that the strain currently circulating in the Americas does not have unusually high infectivity for American *Ae*. *aegypti* as compared to the African strains used in this study. Colonized *Culex* mosquitoes were inefficient vectors. *In vitro* data suggested slower replication and decreased fitness for the currently circulating American strain compared to African strains isolated decades ago. Therefore, viral adaptation to local mosquitoes does not appear, at present, to be driving the current ZIKV outbreak in the Americas.

## Introduction

Zika virus (ZIKV, *Flaviviridae; Flavivirus*), was first isolated from a febrile sentinel rhesus monkey in Uganda in 1947 [1]. Until recently, ZIKV was associated with periodic outbreaks of febrile illness in humans. Prior to 2007, the geographic range was restricted to Africa and parts of Southeast Asia; having been reported from Nigeria [2], Malaysia [3], Sierra Leone [4], Senegal [5], and Indonesia [6]. In 2007, a large outbreak of ZIKV occurred on the island of Yap in Micronesia, which later spread to French Polynesia (2013), New Caledonia (2014), Cook Islands (2014) and Easter Island (2014), before eventually arriving in Brazil in 2014–15 [7–9]. Since arriving in the Americas, local transmission has been reported in a total of 39 countries in the hemisphere as far south as Argentina (http://www.cdc.gov/zika/geo/active-countries.html).

The epidemic of ZIKV in the Americas has been explosive. The factors underlying the rapid spread of the epidemic are likely complex and poorly understood. Based on previous experience with West Nile (WNV) [10] and chikungunya (CHIKV) [11–14] viruses, however, it seems likely that viral factors, and potentially adaptations, that influence vector transmission may at least partially account for the rapid spread of ZIKV. Accordingly, we sought to determine whether the currently circulating ZIKV strain had higher replication rates and/or fitness *in vitro*, or was transmitted more efficiently by American *Aedes (Stegomyia) aegypti* mosquitoes as compared to two different Old World strains. Additionally, we sought to determine the susceptibility of several colonies of *Culex* spp. mosquitoes to the currently circulating American strain. Our studies provide evidence that a strain of ZIKV currently circulating in the Americas (a) does not replicate more efficiently (b) is of decreased competitive fitness and (c) is transmitted efficiently by American *Ae*. *aegypti*, but not *Culex* spp. mosquitoes. Collectively, this work expands our knowledge on transmission of the currently circulating Asian lineage ZIKV in *Ae*. *aegypti* and the potential of divergent ZIKV lineages to be transmitted by American mosquitoes.

## Materials and Methods

### Cells, viruses and mosquitoes

Vero (ATCC CCL-81) and Huh7 (a kind gift from Dr. Richard Kuhn) cells were maintained in Dulbecco’s modified Eagle’s medium (DMEM) containing 10% fetal bovine serum (FBS) and 50μg/mL gentamycin at 37°C with 5% CO_2_. C6/36 cells (ATCC CRL-1660) were maintained in MEM with 10% FBS and 50μg/mL gentamycin at 28°C with 5% CO_2_. Aag2 cells (obtained from Dr. Aaron Brault) were maintained in Schneider’s insect medium with 10% FBS at 28°C.

ZIKV strains PRVABC59 (Accession # KU501215) and MR766 (Accession #AY632535) were obtained from the CDC. Strain 41525 (Accession #KU955591) was obtained from the University of Texas Medical Branch. All viruses were propagated in Vero cells by infection at a MOI of 0.01. Supernatant was harvested 5–6 days post-infection, clarified by centrifugation at 4°C and aliquoted into single use vials before freezing at -80°C. PRVABC59 was isolated in 2015 from an infected human in Puerto Rico and was passaged four times on Vero cells [15]. MR766, the prototype ZIKV strain was isolated from a rhesus macaque in 1947 in the Zika forest in Uganda and was passaged 149 times in suckling mouse brains, followed by three additional Vero passages [1]. The 41525 strain was isolated in Senegal in 1984 from *Ae*. *(Stegomyia) africanus* (Theobald) mosquitoes and was passaged once in *Ae*. *(Stegomyia) pseudoscutellaris* (Theobald) cells, once in C6/36 cells, and 4 times in Vero cells [16].

*Aedes aegypti* were collected from wild populations in Poza Rica, Mexico [17]. Mosquitoes were maintained on calf blood and given 10% sucrose *ad libitum*. Larvae were reared and adults maintained under controlled conditions of temperature (28°C), humidity (70% RH), and light (14:10 L:D diurnal cycle). Experiments involving infectious ZIKV in mosquitoes were performed under BSL3 conditions. The generation of mosquitoes used for these studies was between F11-F13.

For *Culex* infections, three species of laboratory-colony derived mosquitoes were used; *Culex (Cx*.*) quinquefasciatus* Say, *Culex (Cx*.*) pipiens* [L.], and *Culex (Cx*.*) tarsalis* Coquillett. The *Cx*. *pipiens* colony was derived from egg rafts collected in Pennsylvania in 2002. The *Cx*. *tarsalis* mosquitoes were derived from a colony maintained by WK Reisen and collected in California in 1953. The *Cx*. *quinquefasciatus* colony was originally collected in Sebring County, Florida in 1988. Larvae were raised on a diet of powdered fish food. Pupae were allowed to emerge into containers and adult mosquitoes were kept at 26–27°C with a 16:8 light:dark cycle and 70%-80% relative humidity, with water and sucrose provided *ad libitum*.

### *In vitro* replication

Multi- and one-step growth curves were performed on Aag2, C6/36, Huh7, and Vero cells at MOI of 0.01 and 10, respectively. The day before infection, cells were seeded in T25 flasks in triplicate for each virus. Prior to infection, a mock T25 for each cell line was used to count for MOI calculations. Following two hours absorption, virus inoculum was removed, cells were washed with PBS and fresh culture media was added. At each time point (0, 12, 24, 36, 48, 72, 96, 120, 144, and 168 hours), 500μL supernatant was harvested and frozen for later titration. Cells were then supplemented with 500μL fresh media at each time point. Viral titers were determined by plaque assay.

### Vector competence studies

For comparison of fresh and frozen virus on vector competence, Vero cells were infected at a MOI of 0.01 with PRVABC59 4 days prior to a blood-meal of female mosquitoes of the F11-F13 generation (5–7 days post-eclosion). Supernatant was harvested and centrifuged at 3,000xg for 10 min at 4°C to remove any cellular debris. Clarified supernatant was transferred to a clean tube, brought to a 20% concentration of FBS, and aliquoted into two tubes. The first tube, designated “fresh”, was placed at 4°C until further manipulation (~4 hours). The second tube designated “frozen”, was placed at -80°C either for 4 hours or for more than 1 week, and then thawed at 37°C. Mosquitoes were then exposed concurrently to a blood-meal containing 500μL defibrinated calf blood, 5mM ATP (100ul) and 400μL supernatant from either fresh or the two different frozen virus stocks. Following 1-hour feeding, mosquitoes were anesthetized at 4°C and engorged females were separated into new cartons and maintained on sucrose at 28°C. Back-titrations of each infectious blood-meal on Vero cells estimated ZIKV titers to be 2.0x10^6^ PFU/mL and 1.6x10^7^ PFU/mL for fresh and frozen virus, respectively. These values are close to the range of viremia that has been previously reported for hospitalized cases of ZIKV infection [18].

For additional studies using only frozen virus, an infectious blood-meal containing 1.6x10^7^ PFU/mL ZIKV was used. Three experimental replicates were performed with each strain of ZIKV and *Ae*. *aegypti* (Poza Rica). For *Culex* mosquitoes, three replicates were performed for *Cx*. *quinquefasciatus* and one for both *Cx*. *tarsalis* and *Cx*. *pipiens*. Following each infectious blood-meal, back-titration was performed to ensure that virus titers mosquitoes were exposed to during each experimental replicate were comparable. For all studies, on 7 and 14 days post-infection, a subset of mosquitoes was knocked down by cold treatment at 4°C. Legs/wings were removed and transferred into a tube containing 250μL mosquito diluent (1x PBS supplemented with 20% FBS (heat-inactivated), 50 μg/ml Penicillin/Streptomycin, 50 μ/ml Gentamycin, 2.5μg/ml Fungizone) and a stainless steel bead for homogenization. The mosquito proboscis was inserted into a capillary tube containing immersion oil (~5μL) and allowed to salivate for 20 min. Following salivation, mosquito bodies were placed in a separate tube containing mosquito diluent and a bead. The ends of capillary tubes containing oil and saliva were broken off into microcentrifuge tubes containing 100μL mosquito diluent. Mosquito tissues (bodies, and legs+wings) were homogenized at 25 cycles/second for one min using a Retsch Mixer Mill MM400 (Germany) and centrifuged at 15,000xg for 5 min at 4°C. Clarified supernatant was titrated by Vero cell plaque assay to determine the amount of infectious virus present in mosquito bodies. The determination of vector competence was performed essentially as described by Turell et al. [19]. Briefly, the infection rate is defined as the percentage of exposed mosquitoes positive for infectious virus in the body. Dissemination is reported as the percent of exposed mosquitoes that contained infectious virus in their legs and wings, regardless of infection status. The transmission rate is reported as the percent of exposed mosquitoes that contain infectious virus in the saliva, regardless of infection status. Transmission (disseminated) rate (transmission (D) rate) is reported as the percent of mosquitoes with infectious virus in the saliva that were also positive for infectious virus in the legs.

### Competitive Fitness

Competitive fitness was assessed using direct competition assays between each strain of virus in Vero, Huh7, C6/36 and Aag2 cells essentially as previously described [10,20]. Briefly, viruses were mixed 1:1 at a MOI of 0.01 in triplicate and then added to confluent monolayers of cells. After two hours, virus inoculum was removed and fresh medium was added. Supernatant was then harvested at 2 and 3 days post-infection and stored at -80°C for subsequent analysis. RNA was extracted and the region between nucleotides 3649 and 3960 was amplified by RT-PCR. Bands were subjected to Sanger sequencing and the proportion of each virus was assessed by analyzing chromatograms with PolySNP [21].

### Statistical analysis

Comparisons of virus titers in growth curves were performed using two-way ANOVA with Tukey’s correction at each time point. A two-tailed Fisher’s exact test was used to compare rates of infection, dissemination and transmission in vector competence studies. GraphPad Prism 6.0 (La Jolla, CA) was used for all statistical tests and significance was defined as p<0.05.

## Results

### Phylogeny of ZIKV

**Fig 1:**
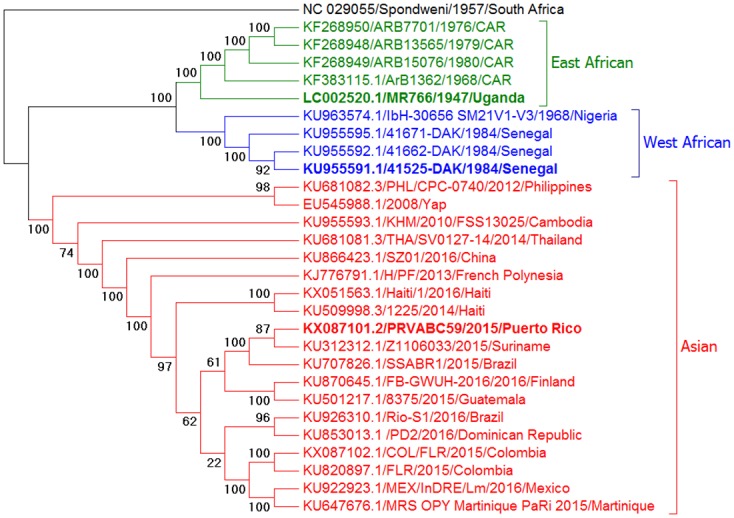
Phylogenetic tree of 29 Zika virus isolates. The tree was generated by Neighbor-Joining methods using the Tamura-Nei genetic distance model (bootstrapped 1,000 times). Similar results were obtained using maximum parsimony or maximum likelihood algorithms. Strains used in this study are shown in bold; distinct lineages are East African (MR766), West African (41525), and Asian (PRVABC59).

Fig 1 depicts the phylogenetic analysis of 29 ZIKV sequences obtained from GenBank using neighbor-joining methods (using the Tamura-Nei genetic distance model [22]) with 1,000 bootstrap pseudoreplicates (maximum likelihood and maximum parsimony analyses were performed and yielded similar tree topography). As previously reported [15], ZIKV sequences separate into three distinct genetic clades; West African, East African, and Asian. For this study, we selected one strain from each clade: strain 41525 from Senegal represented the West African clade, prototype strain MR766 from Uganda represented the East African clade, and the currently-circulating strain from the Americas, PRVABC59 from Puerto Rico, belongs to the Asian clade. The three viruses will be herein referred to as PRVABC59 (Americas), 41525 (West African), and MR766 (East African).

### Growth kinetics *in vitro*

**Fig 2:**
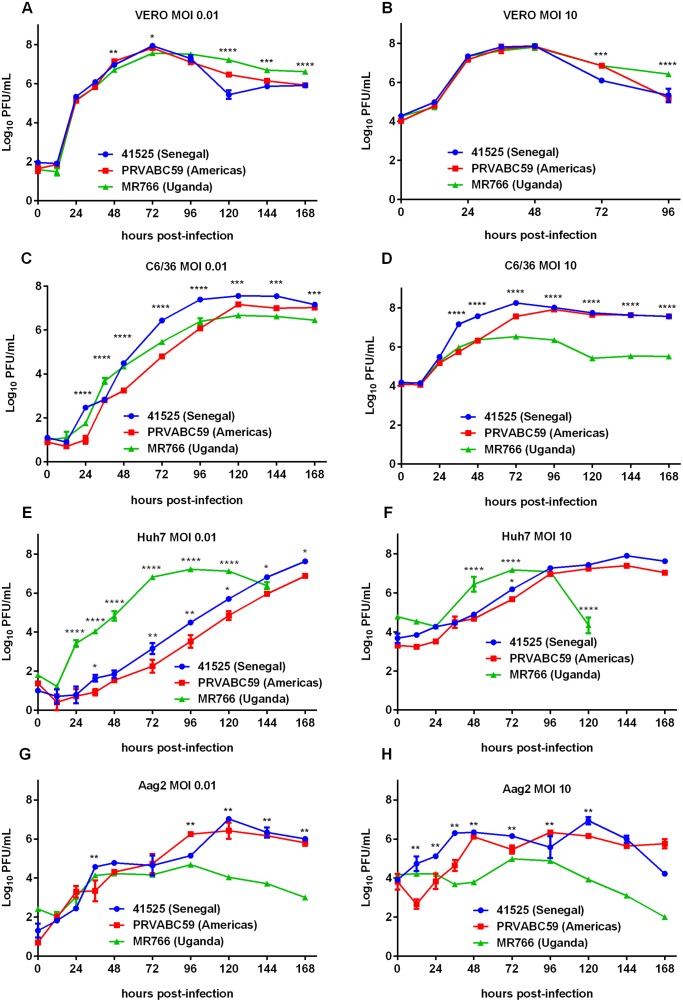
Growth of ZIKV strains in different cell types *in vitro*. Cells were infected with ZIKV strains at a MOI of 0.01 or 10. Following infection, cells were washed and fresh growth medium was added. Supernatant was harvested at the indicated time points for titration via plaque assay. (A) Vero MOI 0.01 (B) Vero MOI 10 (C) C6/36 MOI 0.01 (D) C6/36 MOI 10 (E) Huh7 MOI 0.01 (F) Huh7 MOI 10 (G) Aag2 MOI 0.01 (H) Aag2 MOI 10.

Viral replication was assessed using single- and multi-step growth curves in two mammalian and two mosquito derived cell lines. In Vero cells, which lack functional type-1 interferon (IFN), growth kinetics were similar among the three viruses tested at both MOIs of 0.01 and 10. For MOI of 0.01 at 48h post-infection, significantly higher amounts of virus were observed for PRVABC59 than MR766 but not for 41525 (p = 0.005) (Fig 2a). At peak replication, 72h post-infection, 41525 had a significantly higher titer than MR766, but not PRVABC59 (p = 0.02). At a MOI of 10, no significant differences were observed between any of the strains during peak replication (Fig 2b), however at later time points, 41525 and PRVABC59 titers dropped quickly to significantly lower titers, which was also observed at a MOI of 0.01. In C6/36 cells, which have a deficient RNAi pathway [23], differences in replication were more apparent. Particularly, titers for PRVABC59 were significantly lower at most time points than either 41525 or MR766 after 12 hours and prior to 96h post-infection (Fig 2c). PRVABC59 peak titers subsequently recovered as compared to MR766, which appeared to plateau earlier than the other viruses. At a MOI of 10, 41525 and PRVABC59 also had higher peak titers at 72h than MR766 (p<0.001 for either comparison). In contrast to Vero cells, Huh7 cells, which are IFN competent, displayed vastly different susceptibility to replication by different strains. MR766, which is highly mammalian adapted, replicated to high titers faster at both MOIs of 0.01 and 10 (Fig 2e and 2f). The fall in titer at later time points corresponded with strong CPE caused by MR766 at both MOI. In multi-step growth curves, 41525 also replicated at higher levels than PRVABC59 in Huh7 cells (Fig 2e). In Aag2 cells, which are competent for RNAi, lower levels of replication were observed than in C6/36 cells (Fig 2g and 2h). Peak replication levels were lower for MR766 than either 41525 or PRVABC59, although replication levels were similar for all three strains during the first 72h post-infection at a MOI of 0.01 (Fig 2g). Virus titers appeared to peak around 10^4^ PFU/mL for MR766 at both of the MOI tested, suggesting this virus may have lost some ability to replicate in RNAi competent mosquito cells during mammalian passage, as this was not as apparent in C6/36 cells.

### African ZIKV strains have a direct fitness advantage *in vitro*

**Fig 3:**
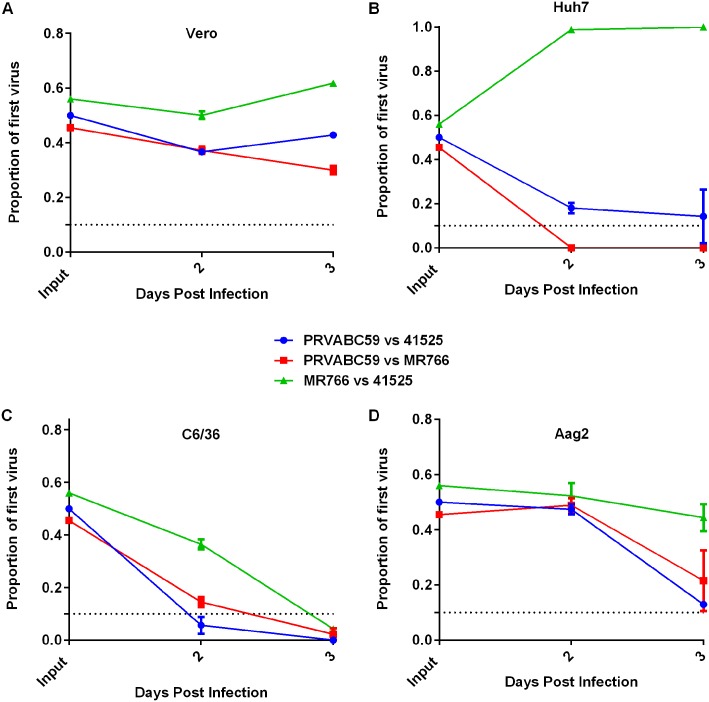
Select strains of East and West African ZIKV have higher fitness *in vitro* than the American strain PRVABC59. Equal amounts of virus (MOI 0.01) was used to infect the indicated cell line. Supernatant was harvested and proportion of each virus was determined by sequencing and analyzing chromatograms. Data are presented as proportion of the first virus listed present at the indicated time point in (A) Vero, (B) Huh7, C6/36 (C), and (D) Aag2 cells.

We have previously shown that direct fitness assays can detect differences that may be unapparent in relative replication assays like growth curves [24]. In competition assays in Vero cells, the proportion of PRVABC59 dropped slightly at 2 and 3 days post-infection, as compared to either of the African strains (Fig 3a). Little change in the proportion of virus was observed during replication between MR766 and 41525. In Huh7 cells, MR766 outcompeted both 41525 and PRVABC59, becoming the dominant virus by day 2 post-infection and essentially pushing the other strains to extinction by day 3 (Fig 3b). In C6/36, PRVABC59 dropped below the limit of detection (~10%) at 3 days post-infection when competed with either other virus (Fig 3c). A similar result occurred with 41525 vs MR766, with the former becoming the dominant virus present in the sample 3 days post-infection. Similarly, in Aag2 cells, PRVABC59 was outcompeted by both African viruses, and by day 3 was only present at low levels, compared to the other two strains (Fig 3d).

### Freezing ZIKV results in decreased infection, dissemination and transmission in *Ae*. *aegypti*

**Fig 4:**
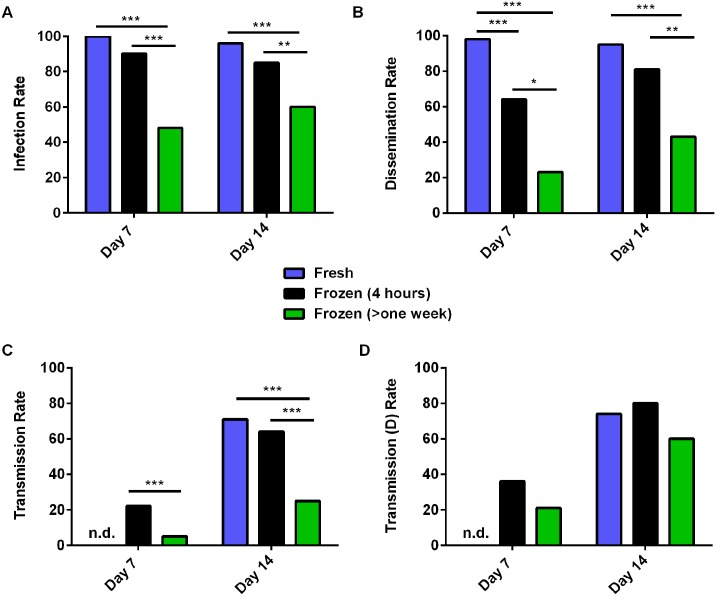
Long term freezing of virus lowers infection rates of *Aedes aegypti* mosquitoes. *Ae*. *aegypti* (Poza Rica strain) mosquitoes were fed an infectious blood-meal containing 1.6x10^7^ PFU of ZIKV (PRVABC59 strain) that was harvested directly from Vero cells (and stored at 4°C for 4 hours) or frozen for 4 hours or more than a week at -80°C. Mosquitoes were held for 7 or 14 days (n = 48 each time point for each group) and then (A) Infection rate (% of mosquitoes with virus in their bodies), (B) Dissemination Rate (% of mosquitoes, regardless of infection status, with virus in their legs), (C) Transmission Rate (% of mosquitoes, regardless of infection status, with virus in their saliva) and (D) Transmission (D) Rate (% of mosquitoes with a disseminated infection, with virus in their saliva) were collected. * Indicates p<0.05, ** p<0.01, and *** p<0.001 by two-tailed Fisher’s exact test. n = 48–144 for each group at each time point. n.d. indicates that samples were not collected for this time-point.

Previous studies have shown that reduced rates of mosquito infection are observed when frozen and thawed virus is used versus freshly harvested virus (i.e. collecting supernatant directly from cells at the peak infection for use in artificial blood-meals without previous freezing) [25–27]. Since little is known about mosquito infections with ZIKV, we first tested whether frozen-thawed virus was equivalent to fresh virus in mosquito infections. Comparing virus that was harvested fresh versus frozen for 4 hours, or frozen for greater than one week, there were clear differences in infectivity to *Ae*. *aegypti* mosquitoes, particularly at early time points (Fig 4a). Seven days post-blood-meal, infection rates were significantly lower for virus that had been frozen for over one week (n = 126–144) but not when it was frozen for just 4 hours (n = 45–48), as compared to fresh virus (n = 48 for both time points) (Fig 4a). However, the use of fresh virus was associated with significantly higher dissemination rates on day 7 than either of the other two groups (Fig 4b). No differences were observed on Day 14 between fresh virus or virus that was frozen for only 4 hours, however all values except transmission (d) rate were significantly lower for virus frozen for longer times as compared to the other two groups (Fig 4c & 4d). Although the infection rates were not significantly different at either time point for mosquitoes exposed to unfrozen virus as compared to those exposed to virus that had been frozen for only 4 hours, infection rates were consistently higher in the mosquitoes exposed to the unfrozen virus. In fact, when infection rates were combined for both day 7 and 14 (which should not change after virus exposure) mosquitoes fed fresh virus have a statistically significantly higher rate of infection than do those fed virus that was frozen for 4 hours (p = 0.01).

### Vector competence of Mexican *Ae*. *aegypti* is highly strain specific

**Fig 5:**
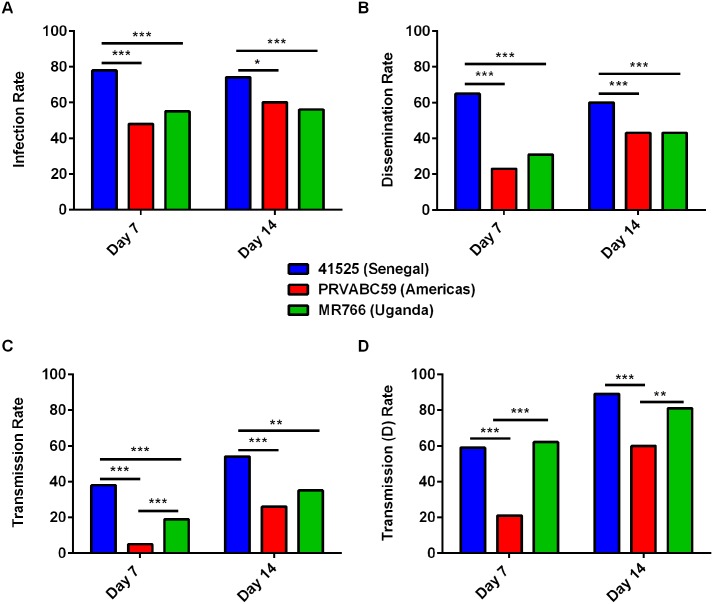
Mexican *Aedes aegypti* have increased vector competence for African strains of ZIKV. Mosquitoes were fed a blood-meal containing 1.6x10^7^ PFU of different ZIKV. Mosquitoes were then held for 7 or 14 days (n = 48 each time point for each group) and then (A) Infection rate (% of mosquitoes with virus in their bodies), (B) Dissemination Rate (% of mosquitoes, regardless of infection status, with virus in their legs), (C) Transmission Rate (% of mosquitoes, regardless of infection status, with virus in their saliva) and (D) Transmission (D) Rate (% of mosquitoes with a disseminated infection, with virus in their saliva) were collected. * Indicates p<0.05, ** p<0.01, and *** p<0.001 by two-tailed Fisher’s exact test. n = 126–144 for each group at each time point.

To assess the vector competence of Mexican *Ae*. *aegypti* for different strains of ZIKV, mosquitoes were fed an artificial blood-meal and tissues harvested 7 and 14 days later. Infection rates were significantly higher on days 7 and 14 for mosquitoes exposed to strain 41525 (n = 126 for both time points) as compared to groups exposed to either MR766 or PRVABC59 (n = 144 for both groups, for both time points) (Fig 5a). Dissemination of ZIKV on day 7 and day 14 post-infection was significantly lower for MR766- or PRVABC59-infected mosquitoes as compared to mosquitoes infected with 41525 (Fig 5b). Furthermore, after 7 and 14 days following blood-meal, the transmission rate was significantly higher for either African virus, as compared to PRVABC59 (Fig 5c). Additionally, the transmission (d) rate was significantly higher for strain 41525 as compared to PRVABC59 at both time points (Fig 5d), indicating this virus strain may have a fitness advantage in these mosquitoes.

### Colonized *Culex* mosquitoes are refractory to ZIKV PRVACB59 infection

Mosquitoes in the genus *Culex* are also important arbovirus vectors, particularly of other flaviviruses; WNV [10], Japanese encephalitis virus [28] and St. Louis encephalitis virus [29]. Therefore, we sought to determine whether the epidemic American strain PRVABC59 could be efficiently transmitted by several species of *Culex* mosquitoes. Neither fresh nor frozen ZIKV efficiently infected any of the three species tested (*Cx*. *quinquefasciatus*, *Cx*. *tarsalis*, and *Cx*. *pipiens)* (Table 1). Except for infectious virus detected in one *Cx*. *quinquefasciatus* mosquito exposed to frozen ZIKV, no mosquitoes became infected (*Cx*. *quinquefasciatus* n = 144, *Cx*. *tarsalis* n = 20, and *Cx*. *pipiens* n = 48).

**Table 1:** Infection of *Culex* spp after peroral exposure to ZIKV PRVABC59.

Species	Bloodmeal Titer (PFU/mL)	Fresh or Frozen	Day 7	Day 14
*Cx*. *quinq*	1.6x10^7^	Frozen (1 week)	1/48	0/48
*Cx*. *quinq*	1.6x10^7^	Frozen (1 week)	0/48	0/48
*Cx*. *quinq*	5.0x10^6^	Fresh	0/48	0/48
*Cx*. *pipiens*	5.0x10^6^	Fresh	0/48	0/48
*Cx*. *tarsalis*	5.0x10^6^	Fresh	ND	0/20
*Ae*. *aegypti*	1.6x10^7^	Frozen (1 week)	67/141	86/144
*Ae*. *aegypti*	1.6x10^7^	Fresh	48/48	43/45

## Discussion

The vector competence of mosquitoes from the Americas for transmission of ZIKV is not well defined, despite the ongoing explosive outbreak. This study aimed to define the growth kinetics, relative fitness and vector competence of Mexican *Ae*. *aegypti* mosquitoes for several strains of ZIKV, including an Asian lineage strain isolated during the ongoing ZIKV outbreak in the Americas. In addition, we sought to examine the effect of freezing ZIKV prior to performing mosquito experiments. Finally, we tested infectivity of ZIKV in several species of *Culex* mosquitoes.

While some differences in replication kinetics were observed in mammalian cells between strains, the most obvious differences were noted in mosquito cells, particularly C6/36, in which both African strains reached significantly higher titers at many time-points in both one- and multi-step growth curves. These data were further supported by vector competence results, as PRVABC59 had lower infectivity in *Ae*. *aegypti* mosquitoes that were originally collected in Poza Rica, Mexico as compared to the other two virus strains. This observation was surprising, as the population of *Ae*. *aegypti* used is expected to be closely related to the populations that vector ZIKV strain PRVABC59 in the current outbreak. Furthermore, competitive fitness data *in vitro* strengthen the conclusion that both African strains tested here were more fit in mosquitoes than the Asian lineage isolate, as the latter became extinct in competition with either of the African viruses *in vitro*.

The effect of freezing ZIKV on vector competence is notable and should be considered when designing mosquito infection experiments. It is unclear what factors are playing a role in this loss in infectivity as short-term freezing did not appear to have the same effect as more extended storage at -80°C. Previous reports have suggested that virion morphology may be altered during freezing, which causes a specific reduction in infectivity in mosquito cells [30]. It has also been shown recently that the flavivirus NS1 protein is critical for mosquito infection, down-regulating host immune responses which results in increased infection rates [31]. Stability of NS1, which exists in dimeric and hexameric forms, has been shown to rely on surface glycosylation, which could be affected during long term freezing [32]. Loss of an N-linked glycan on NS1 was shown to reduce interaction with mammalian complement proteins, indicating interactions with mosquito proteins may also be compromised. Future studies comparing the mosquito immune response with fresh or frozen virus should be performed to assess what role NS1 may be playing in this loss in infectivity. Supplementation of exogenous NS1 may be sufficient to regain levels of mosquito infection with frozen virus, thus allowing the generation of long-term frozen stocks to be used more consistently.

In all measures tested here (growth kinetics, mosquito infections, competition assays, and genome-to-PFU ratios), the African strains displayed either equal or greater fitness than the currently circulating American strain PRVABC59 of the Asian genotype. This result was particularly surprising for MR766, which has been extensively passaged in mice and, as a result, may have been expected to lose the ability to replicate efficiently in intact mosquitoes [33,34]. The reason for this is unknown and these differences should be determined by in-depth phylogenetic analyses using a reverse genetics approach to assess the relative importance of different regions of the virus genome in viral fitness in mosquitoes and vertebrate hosts. One limitation of this study is that a single strain from each ZIKV lineage was used in these studies (and MR766 was highly passaged); the relationships of these strains to wild-type strains remains to be determined, but genetic differences among sequenced members of each clade appear to be minimal [15]. Nonetheless, the high relative fitness and mosquito transmissibility of the African strains (which were more highly passaged compared to PRVABC59) suggests that passage has not resulted in loss of vector infectivity. Based on the data presented here, it appears that the explosive nature of the current outbreak may not be solely due to viral genetic factors, but rather other factors such as a naïve human population, underreporting in Africa and Asia, increased global travel, poor hygiene, and an abundance of sufficiently competent, highly anthropophilic mosquito vectors. It is possible that a convergence of entomological, viral and human factors are responsible for the current ZIKV outbreak. Determining why large ZIKV outbreaks are rare, non-existent or go unreported in Africa may provide clues on factors involved in the current outbreak. Additional studies are necessary to identify specific viral residues that may be important in adaptation to transmission in the Americas by comparing them to residues in previously circulating strains in Asia. It is conceivable that one or several mutations allow for increased replication in humans or certain mosquito species; as has previously been seen with chikungunya virus [12,14,35]. In addition, as an RNA virus, the ZIKV strain circulating in the Americas is expected to adapt further, therefore continued surveillance of circulating strains and additional vector competence work with these strains is necessary. Furthermore, active surveillance is needed for ZIKV in Africa, as very little is known about sero-prevalence or the currently circulating African strains. The African strains of ZIKV used in this study were isolated decades ago and may not represent the currently circulating genotypes. If the currently circulating strains of ZIKV have an advantage in American mosquito species or populations as compared to viruses in the Asian lineage, it is possible that introduction would result in increased spread and possibly more disease.

Previous studies examining vector competence of *Aedes* mosquitoes for ZIKV have produced conflicting results. Wong et al. [36] showed that *Ae*. *(Stegomyia) albopictus* Skuse mosquitoes from Singapore were highly susceptible to ZIKV strain MR766, with 100% of mosquitoes having infectious virus in the saliva by day 14 post-blood-meal. Following the Yap island outbreak, Ledermann et al. [37] showed high infection rates and modest dissemination rates in *Ae*. *(Stegomyia) hensilli* Farner mosquitoes using ZIKV strain MR766 8 days after infectious blood-meal. In contrast, Diagne et al. [38] reported very low dissemination and almost no transmission of several strains of ZIKV (including MR766 and several strains from Senegal) in Senegalese *Aedes* mosquitoes. Given the role of Ae. aegypti in the current outbreak in the Americas, it was interesting that low transmission was observed among domestic or sylvatic *Ae*. *aegypti* from Senegal when mosquitoes were infected with local strains of ZIKV. Finally, using an Asian genotype strain of ZIKV from New Caledonia and several populations of *Ae*. *aegypti* and *Ae*. *albopictus* from the Americas, Chouin-Carneiro et al. [39] showed high levels of infection and moderate and low levels of dissemination and transmission, respectively, similar to the results reported here for ZIKV strain PRVABC59. While these studies involved different strains of ZIKV and different species and collections of mosquitoes from diverse geographic locations, these data demonstrate the variability that can be observed in vector competence studies, and the dependency on the particular virus and mosquito strains evaluated and experimental parameters employed. Mosquitoes used in this study were of the F11-F13 generation and thus it is possible that these results do not reflect the actual transmission phenotype of field mosquitoes. Although studies using such mosquitoes are critical, our goal here was mainly to assess whether the explosive nature of the current outbreak was attributable to high transmissibility and/or replicative capacity of the virus strain in its current environment. Further studies should examine the refractory nature of Senegalese mosquitoes to local ZIKV strains and investigate the specific determinants of vector competence of American *Ae*. *aegypti* mosquitoes for transmitting outbreak strains of ZIKV from the Asian lineage as compared with either of the African lineages. It is possible that different American populations of *Ae*. *aegypti* are more susceptible to the ZIKV strain currently circulating in the Americas and this is facilitating the current outbreak. Bennett et al. showed that vector competence can vary greatly among *Ae*. *aegypti* populations in Mexico.[40]

In the laboratory colonies of *Culex* mosquitoes tested here, we observed very little to no infection with the epidemic strain of ZIKV, PRVABC59. Several reports have now shown *Culex* spp. to be refractory to infection [41–45], including one field report [46]. Only one report has shown *Culex* to be susceptible to ZIKV infection [47]. This is the first report of VC of *Cx*. *tarsalis* mosquitoes with ZIKV. The laboratory colonies used here; despite extensive time in colony, have been used at length for WNV experimentation with high infection, dissemination and transmission rates, providing evidence that they are not generally refractory to flavivirus infection [48]. Further, field populations of *Cx*. *pipiens*, *Cx*. *quinquefasciatus*, and *Cx*. *tarsalis* are not nearly as anthropophilic as *Ae*. *aegypti* (although *Cx*. *quinquefasciatus* may readily feed on humans in some environments [49]), taking a large proportion of blood meals from birds and other non-human vertebrate hosts [50,51]. Therefore, it is unlikely that these species will play a major role in ZIKV transmission. This also predicts that it is unlikely that ZIKV circulates in epizootic cycles involving birds, at least in North America.

Our data demonstrate that the three strains of ZIKV tested here can replicate and disseminate in *Ae*. *aegypti* mosquitoes originally collected from Mexico. Interestingly, the two strains of ZIKV from Africa displayed higher rates of infection, dissemination and transmission in these mosquitoes than the American strain of the Asian lineage that is currently circulating. This was unexpected, as MR766 has a long passage history in mice, which is thought to lead to fitness costs in mosquitoes [33,34]. This provides additional evidence that the ZIKV strain currently circulating in the Americas does not have higher fitness in American mosquitoes. This finding suggests the possibility that introduction of other lineages of ZIKV could result in autochthonous transmission. This is significant, because the extent of cross-protection between ZIKV genotypes is unknown, and new reports have shown that antibody-dependent enhancement (ADE) can occur through ZIKV interaction with anti-dengue antibodies [52]. Further experiments should be performed to assess the protection conferred from one genotype of ZIKV to another. Taken together, our data show that American *Ae*. *aegypti*, but not *Culex*, mosquitoes are susceptible to several strains of ZIKV.
